# Monitoring the intracellular calcium response to a dynamic hypertonic environment

**DOI:** 10.1038/srep23591

**Published:** 2016-03-23

**Authors:** Xiaowen Huang, Wanqing Yue, Dandan Liu, Jianbo Yue, Jiaqian Li, Dong Sun, Mengsu Yang, Zuankai Wang

**Affiliations:** 1 Department of Mechanical and Biomedical Engineering,City University of Hong Kong, Hong Kong, China; 2 Department of Biomedical Sciences,City University of Hong Kong, Hong Kong, China; 3 Key Laboratory of Biochip Technology, Biotech and Health Centre,Shenzhen Research Institute of City University of Hong Kong, Shenzhen, China

## Abstract

The profiling of physiological response of cells to external stimuli at the single cell level is of importance. Traditional approaches to study cell responses are often limited by ensemble measurement, which is challenging to reveal the complex single cell behaviors under a dynamic environment. Here we report the development of a simple microfluidic device to investigate intracellular calcium response to dynamic hypertonic conditions at the single cell level in real-time. Interestingly, a dramatic elevation in the intracellular calcium signaling is found in both suspension cells (human leukemic cell line, HL-60) and adherent cells (lung cancer cell line, A549), which is ascribed to the exposure of cells to the hydrodynamic stress. We also demonstrate that the calcium response exhibits distinct single cell heterogeneity as well as cell-type-dependent responses to the same stimuli. Our study opens up a new tool for tracking cellular activity at the single cell level in real time for high throughput drug screening.

The study of cellular physiological responses to external microenvironments with high spatial and temporal resolution is of importance for probing cell signaling and function^1^,^2^,^3^,^4^,^5^. Exposed to different stimuli, various cellular responses will be activated such as the cell shrinkage (volume decrease)^6^,^7^, gene expression^8^ and underlying ion movement (potassium, sodium and calcium)^9^,^10^,^11^. In particular, the calcium signaling of most of cells is mediated by various molecular pathways, e.g., inositol trisphosphate (IP3), adenosine 5′-triphosphate (ATP), prostaglandin E2 (PGE2), and nitric oxide (NO). IP3 can lead to a rapid release of calcium stored in the ER via binding to the ER membrane receptor. After the cytosolic calcium concentration is elevated to a critical level by intra/extracellular sources, the depleted intracellular calcium stores tend to recover their calcium reservation to original level and become ready for the next release of calcium^12^,^13^,^14^. Traditional approaches to study the intracellular calcium response typically involve the exposure of a group of cells to external environments. Such ensemble measurement could not reflect the heterogeneity of individual cells in transient response subject to dynamically changing environments^15^. Different from ensemble measurements, single cell analysis is capable of revealing the complex, highly orchestrated physiology of individual cellular processes^16^,^17^. Although patch clamp could be used to detect the cellular response to external conditions in real-time, the immobilization of single cells by patch clamp is rather labor-intensive and time-consuming, which is inherently not amenable for high-throughput analysis^18^. Flow cytometry could achieve high-throughput single cell detection, but it is an end-point analysis and the dynamic change in cellular activity could not be monitored in real-time^16^,^19^. Microfluidic technologies offer advantages of high-throughput single-cell measurements of cellular responses^20^,^21^,^22^. One promising approach is to trap single cell in large microwell arrays, which have been demonstrated for drug screening, toxicology, and fundamental cell biology^23^,^24^,^25^,^26^.

In this work, we report the monitoring time resolved intracellular calcium response to dynamic hypertonic conditions using a simple microwell-based microfluidic device. The hypertonic condition is regulated by the liquid evaporation which serves to mimic dynamic osmolality decrease that cells might encounter in vivo. Interestingly, a substantial elevation in the intracellular calcium signaling is found in both suspension cells (human leukemic HL-60 cells) and adherent cells (lung cancer A549 cells) at certain hypertonic condition, though the intracellular calcium response exhibits obvious cell-type-specific difference as well as pronounced single cell heterogeneity. We determine that this sharp rise in the calcium concentration is due to the hydrodynamic stress stimulus resulting from the exposure of cell to the air-liquid interface. We envision that the simple platform reported here may open up a new avenue for the real-time monitoring of cellular responses to dynamic stimuli with high throughput and precision at the single cell level.

## Results and Discussion

**Figure 1:**
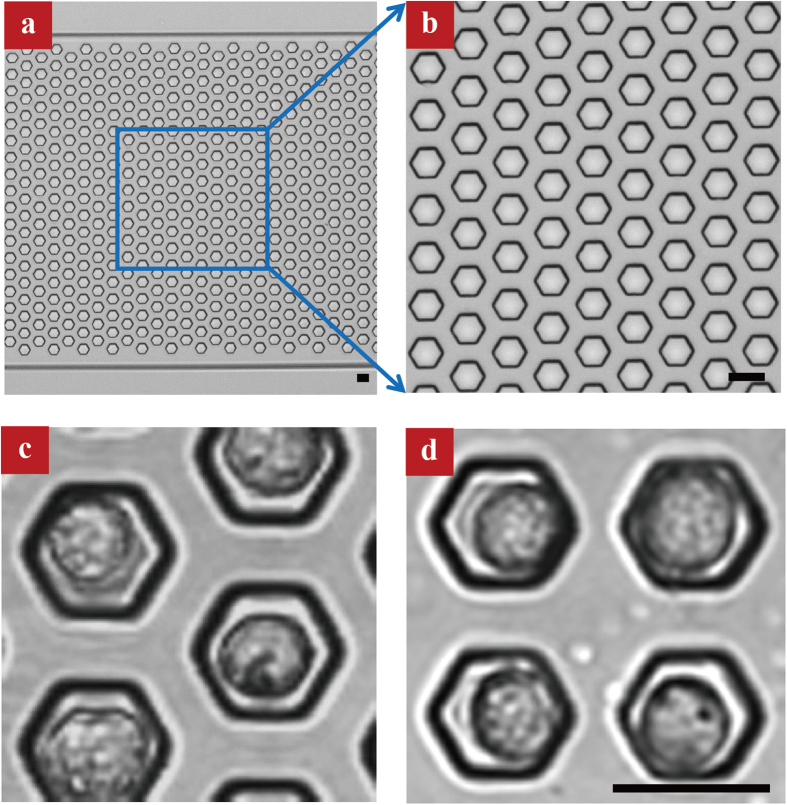
Optical images of patterned microwell arrays with single cell entrapment. **(a**,**b)** Photograph of as-fabricated microwell arrays in a honeycomb lattice. The diameter, depth and center-to-center spacing of microwell is 20, 27, and 40 μm, respectively. **(c**,**d)** Optical images of microwells in a honeycomb or square lattice with single cell entrapment. The scale bar in all the figures is 20 μm.

Figure 1a shows the optical image of as-fabricated microfluidic chip using the soft lithography process. The microchannel comprises patterned microwell arrays. The diameter, depth and center-to-center spacing of microwells are 20, 27, and 40 μm, respectively, corresponding to a volume of 7 nL. In our experiment, microwell arrays are arranged either in a honeycomb (Fig. 1c) or in a square (Fig. 1d) lattice. To trap a single cell into individual microwell, the Ca^2+^-free HBSS was first introduced into the microfluidic chip using vacuum. Then, we introduced 5 μL Fluo 3-AM stained HL-60 cell suspension (2 × 10^8^ cells/mL) onto the chip, followed by incubation for 25 min to allow cells to sedimentate to the bottom of the microwells. Finally, redundant cells were flushed with fresh calcium-free HBSS.

**Figure 2:**
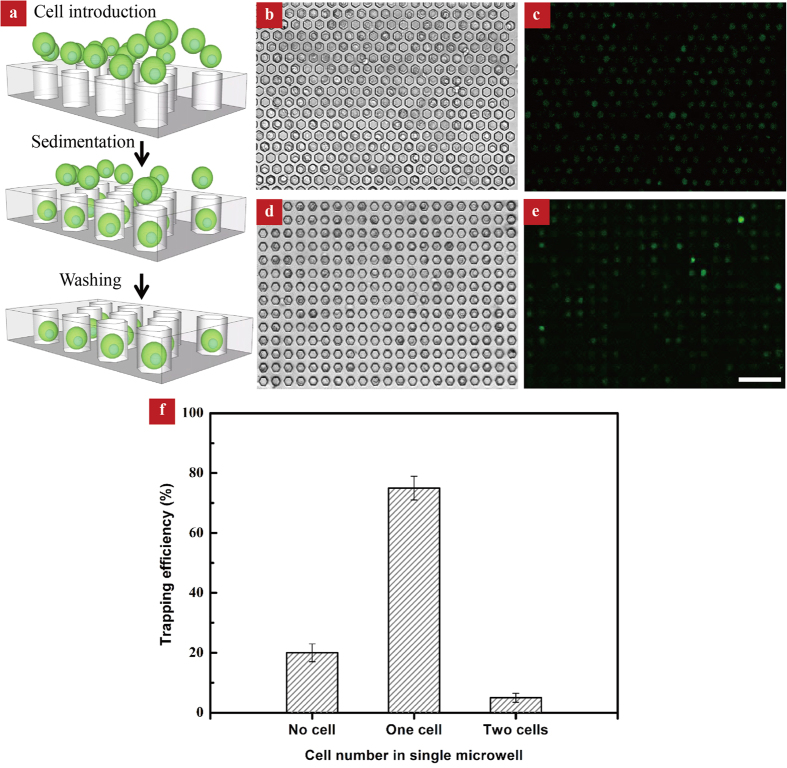
Cell trapping procedure and analysis. **(a)** Schematic depiction of the cell trapping procedure. **(b**,**c)** Bright-field and fluorescence images showing cells trapping in a honeycomb lattice. **(d**,**e)** Bright-field and fluorescence images showing cells trapping in a square lattice. The scale bar is 100 μm. (**f**) Characterization of cell distribution in microwells.

We found that the cell trapping efficiency, or the number of trapped cells relative to the total wells, is mainly dependent on both the cell seeding density and the sedimentation time. Notably, there is no discernible difference in the cell trapping efficiency between microwell arrays with different lattices (honeycomb or square). For a cell seeding density of 2 × 10^9^ cells/mL with a sedimentation time over 25 min, a trapping efficiency up to ~85% can be achieved. However, the use of higher cell seeding density also increases the possibility of trapping multiple cells in a single microwell. When the seeding density is reduced to 2 × 10^8^ cells/mL, the trapping efficiencies in the two types of microwell arrangements are similar (~ 80%), with ~75% wells containing single cell and ~5% microwells containing two cells (Fig. 2b, d and f). The number of cells can be easily determined by analyzing the bright-field images since the cells maintain an integral morphology prior to the evaporation experiment. In our experiment, we chose the cell seeding density of 2 × 10^8^ cells/mL and incubation time of 25 min.

**Figure 3:**
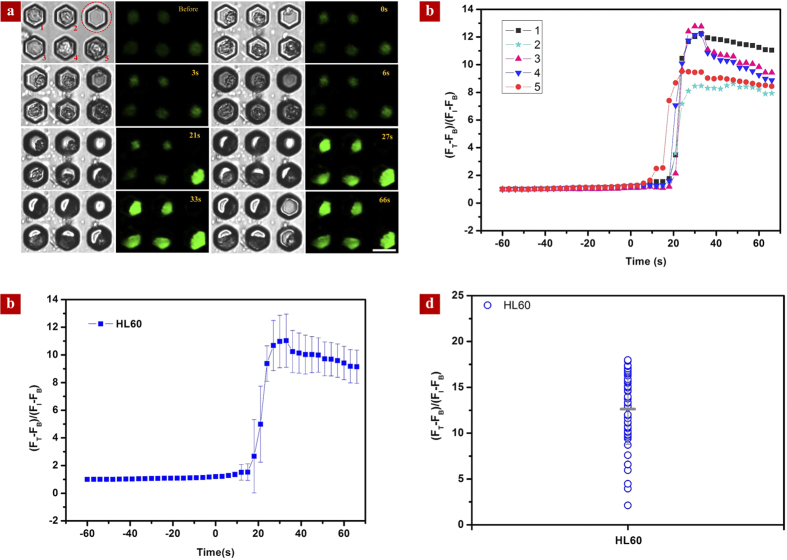
Intracellular calcium response to dynamic hypertonic conditions. **(a)** Bright-field (left) and fluorescence (right) images showing the time-resolved evolution of cell morphology and fluorescence intensity, respectively. The microwells trapped with a single cell are numbered and an empty microwell circled by the dotted red line is used as control. During the early stage of evaporation (from −60 s to 0 s), there is no remarkable change in the fluorescence signal. Around 21 s, a significant enhancement in the fluorescence signal is observed. **(b)** The variation of the enhancement factor in the fluorescence signal as a function of time for 5 cells. **(c)** The time-resolved variation of the enhancement factor in the fluorescence signal based on the average of 50 HL-60 cells. **(d)** The individual fluorescence signal responses of 50 HL-60 cells at ~66 s suggest distinct cell heterogeneity.

**Figure 4:**
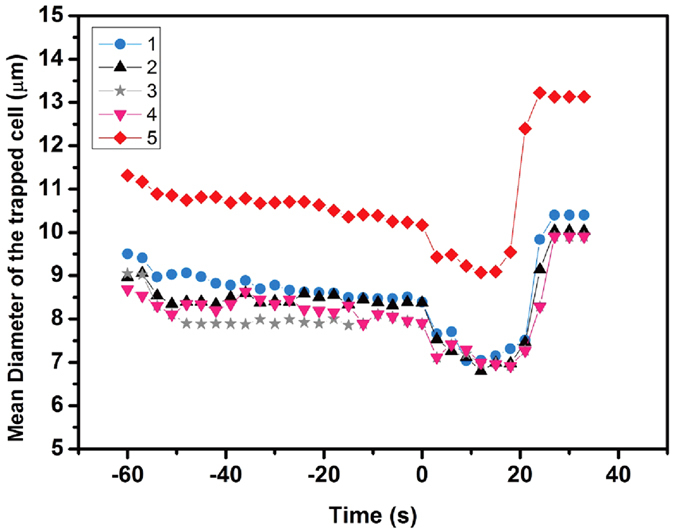
The change of cell diameter as a function of time. From 0 s to 18 s, the decrease of cell diameter indicated the cell shrinkage induced by the increasing hypertonic concentration but diameters of cells increased dramatically to constant values.

Next, we examined real-time intracellular calcium signaling in response to dynamic hypertonic conditions. We chose the human leukemic HL-60 cell line, which were derived from a 36-year-old woman with acute promyelocytic leukemia at the National Cancer Institute. Figure 3a shows the selected bright-field (left) and fluorescence (right) images of cells throughout the evaporation process, respectively. At the beginning, the evaporation is primarily occurring outside the microwell and the air-liquid interface remains flat. We did not observe any marked change in the fluorescence signal, although there is a size reduction of ~1.1 μm in the average diameter as a result of the osmotic effect (Fig. 4). As the liquid inside the microwell evaporates with the time progression, the air-liquid interface becomes curved. As a result of light diffraction, the rim of the microwell becomes darker. We denote the critical time for the rim of microwell to emerge a dark feature as zero. In the following stage until ~21 s, the average diameter of cells decreases to 7.5 μm due to the osmotic effect. Despite the continuous size reduction, there is no obvious change in the fluorescence intensity, suggesting that the intracellular calcium concentration is not sensitive to the increasing hypertonic concentration. However, an abrupt elevation in the fluorescence intensity was observed after 21 s. Close inspection of the cell morphology reveals that the cell is also associated with a concurrent increase in size. To quantify the profound augmentation in the fluorescence intensity, we defined the real time fluorescence intensity (F_T_) relative to the initial fluorescence signal (F_I_) as the enhancement factor, which can be expressed as (*F*_*T*_ − *F*_*B*_)/(*F*_*I*_ − *F*_*B*_) × 100%, where F_B_ is the background fluorescence intensity of the chip. The enhancement factor as a function of time was plotted in Fig. 3b. Note that after reaching the maximum value of ~13 at ~30 s, the enhancement factor remains almost saturated. Interestingly, the intracellular calcium level does not fully recover to the previous baseline, which might be due to the occurrence of cell death in high hypertonic conditions. Moreover, we found that the calcium response of individual cells exhibits distinct cell heterogeneity, as indicated by the large deviation in the enhancement factor of five cells (Fig. 3b). To demonstrate the utility of our platform for the large scale cell analysis, we also plotted the average time-resolved calcium responses based on the statistics of 50 cells in Fig. 3c as well as the individual calcium responses at ~66 s in Fig. 3d, both of which confirm our conclusions on the emergence of strong calcium response at a proper hypertonic condition and distinct single cell heterogeneity (Fig. 3d).

**Figure 5:**
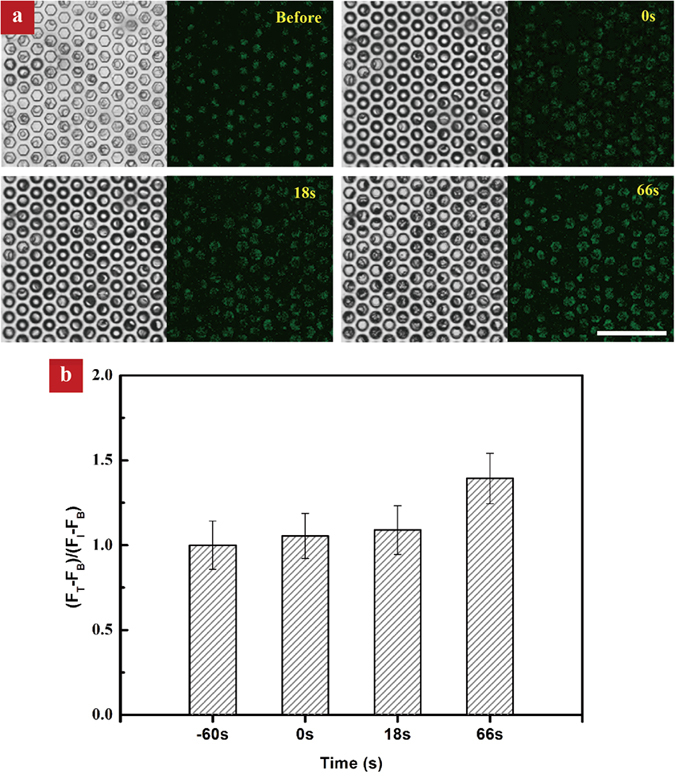
The effect of the fluorescence dye enrichment on the enhancement factor. **(a)** Control experiment based on Calcein AM stained HL-60 cells shows that there is no remarkable change in the fluorescence intensity during the evaporation process. **(b)** The small enhancement factor obtained in the control experiment suggests that the sudden increase in the calcium response in our experiment is not caused by the dye enrichment itself.

To rule out that the elevation in the fluorescence intensity is due to the enrichment of fluorescence dye during evaporation, we conducted a control experiment using Calcein AM instead of Fluo 3-AM to stain HL-60 cells. Calcein AM is a cell-permeate dye widely used to determine cell viability in most eukaryotic cells and the fluorescence intensity would not be affected by the calcium level in cells. As indicated in Fig. 5a and b, the fluorescence intensity maintains almost constant and the maximum enhancement factor is about 1.39. Such an increase in the fluorescence intensity is one order of magnitude smaller than that observed in the case of Fluo 3 stained cells, revealing that the measured increase in the cellular calcium signaling (Fig. 3d) is not caused by the dye enrichment.

**Figure 6:**
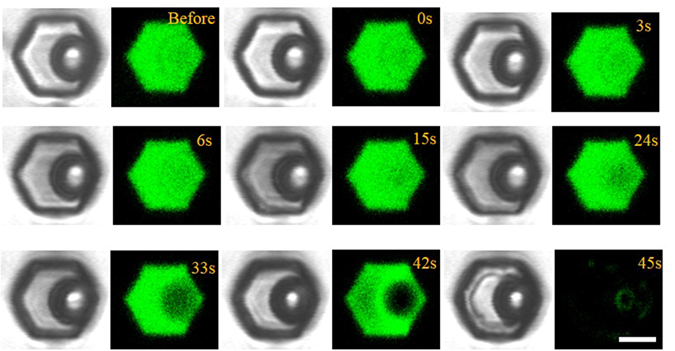
Determination of the critical time for cell to expose to the air/liquid interface. Bright-field (left) and fluorescence (right) images showing the evaporation process of microbead suspension with the calcium-free HBSS containing fluorescent FITC. The critical time for the microbead to expose to the liquid/air interface is at ~15 s. Such a time is comparable to the critical time point at which we observed an abrupt increase in fluorescence intensity and cell elongation (Fig. 3b).

To elucidate the underlying reason for the elevation in the calcium signaling, we conducted a control experiment using microbeads to replace cells. The diameter of microbeads is similar to the cells. We first prepared the microbead suspension with the calcium-free HBSS containing fluorescent FITC. After trapping the microbead in microwell using the procedure described above, we captured bright-field and fluorescence images every 1.5 s. Since there is no fluorescence signal from the microbead itself, the height of the liquid solution can be easily determined by examining the variations of the fluorescence intensity in the microbead and the liquid solution. As shown in Fig. 6, the critical time for the microbead to expose to the liquid/air interface is at ~15 s, which is relatively smaller than the critical time point at which we observed an abrupt increase in fluorescence intensity and cell elongation (Fig. 3b). This mild difference in the critical time point might be due to the fact that cells can deform themselves and release water from intracellular environment. Since there is no calcium influx from extracellular medium, the increase of intracellular calcium concentration is ascribed to the release of intracellular calcium stores. Previously, it has been reported that when cell is exposed to the air interface, the moisture escaping from the cellular membrane can exert surface tension forces up to 320 tons/in^2^ 27. Moreover, although some cells can release calcium under abrupt increase of osmolarity, most cells are less active in hypertonic environment in terms of calcium signaling^28^. Thus, we propose that the cell elongation and concurrent increase in the calcium signaling was due to the large cellular hydrodynamic stress resulting from the exposure of cells at the air/liquid interface.

**Figure 7:**
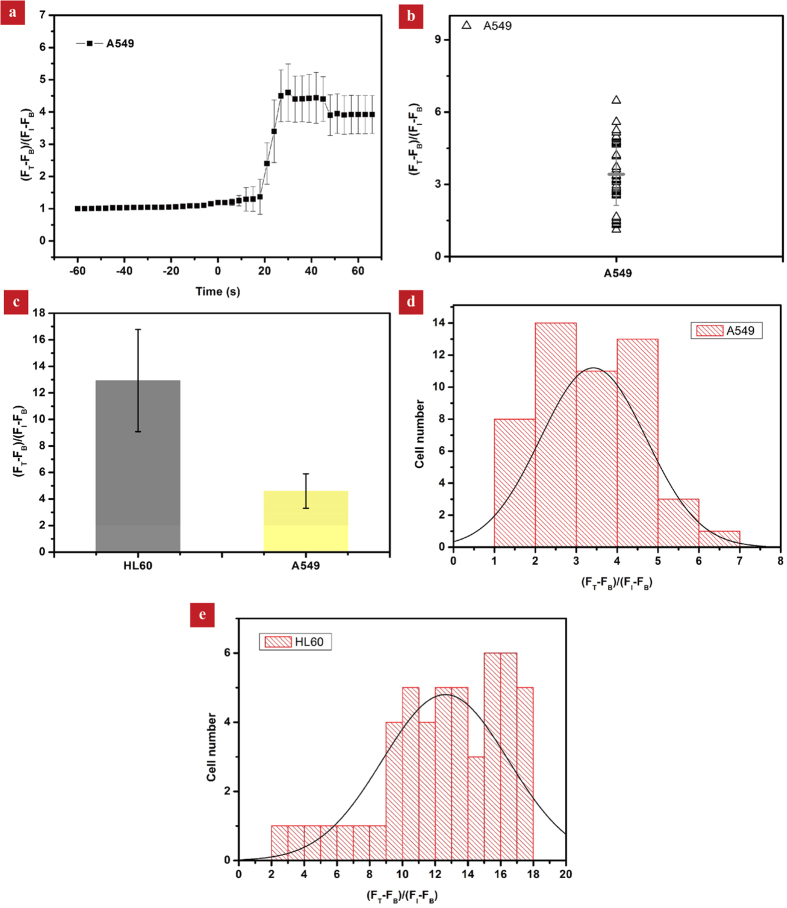
(a)The variation of the fluorescence signaling of A549 cells (n = 50) as a function of time based on the average of 50 cells. The enhancement factors at ~24 s and ~66 s are 4.6 and 3.9, respectively, both of which are much smaller than that in the case of HL-60 cells. **(b)** The variation of individual cell calcium responses at ~66 s. (**c**) The comparison of the magnitude of calcium peaks for two cell lines (HL-60 and A549). (**d**,**e**) The statistical distribution of the cellular calcium signal amplification for HL-60 (n = 50) and A549 (n = 50) cells at 66 s, suggesting a notable cell-type-specific differences and single cell heterogeneity.

To demonstrate the general and robust nature of our platform, we also investigated the calcium response of adherent lung cancer cells (A549 cell line) subject to increasing hypertonic conditions. Under the same experimental condition, the enhancement factors at ~24 s and ~66 s are 4.6 and 3.9, respectively, both of which are much smaller than that in the case of HL-60 cells (Fig. 7a–c). We suspect that the smaller response in A549 is due to its robust cytoskeleton network which offers a better mechanical support, though the origin for the difference in the mechanical stimulation remains to be investigated. Moreover, we also plotted the number distributions of both HL-60 and A549 cells as a function of cellular calcium signal amplification at 66 s (Fig. 7d and e). These results clearly suggest a notable cell-type-specific difference and single cell heterogeneity in the same cell type.

## Conclusion

In summary, we developed a simple microfluidic platform to investigate the calcium response to dynamic hypertonic conditions in real time. This method is simple to operate, and deviates the need of complex dilution process to achieve different hypertonic conditions. We found that there is a sudden increase in the intracellular calcium releasing from intracellular calcium store at a certain hypertonic condition, which results from the exposure of cell to a large hydrodynamic stress. We further show that the calcium responses to hypertonic conditions exhibit apparent cell-type-specific differences and single cell heterogeneity. Since the simple and high-throughput nature of our device, as well as the novel findings of the intracellular calcium responses in this experiment, we assert that this method will be beneficial in developing microfluidic device applications for future research on cellular response to various stimuli. In the future, a well-controlled evaporation process allows us to modulate the hydrodynamic stress which allows us to probe the intracellular calcium response to a wide range of mechanical stimuli. We envision that the platform can allow for simultaneous measurement of other molecules capable of probing dynamic signal pathways such as kinase activity and membrane potential.

### Methods Materials and reagents

The Sylgard^®^ 184 polydimethyl siloxane (PDMS) kit was purchased from Dow-Corning. AZ 50XT Photoresist and developer were from AZ Electronic Materials (Somerville, NJ, USA). Calcein-AM was from Sigma Aldrich (MO, USA), cell culture medium and fetal bovine serum (FBS) were from the Gibco Invitrogen Corporation (CA, USA). Fluo 3-AM (Molecular Probes, Eugene OR, USA) stock solution (6 mM) was prepared in dry dimethyl sulfoxide (Sigma Chemical CO., St Louis, MO, USA) and diluted by fresh RPMI-1640 medium without FBS to a final concentration of 5 μM. Fluorescein-5-Isothiocyanate (FITC) was purchased from Sigma Chemical CO. (St Louis, MO, USA). Analytical reagent grade solvents and other chemicals were purchased from local commercial suppliers, unless otherwise stated. All solutions were prepared using ultra purified water supplied by a Milli-Q system (Millipore).

### Device fabrication

The microfluidic device was fabricated using soft lithography method^29^,^30^,^31^. Briefly, a photomask was first designed and fabricated on a transparent film (Kernel Electronics Co., Ltd., Guangzhou, China). Then, the pattern from the photomask was transferred into a Su-8 substrate by photolithography and the mold consisted of pillar arrays. After pouring the mixture of PDMS base and curing agent (10:1) onto the mold, degassing and baking at 80 °C for 60 min, we peeled the PDMS from the mold, forming a PDMS replica with microwells. Finally, after opening the inlet and outlet from, the PDMS replica was bonded onto a glass slide.

### Cell culture and staining

Human promyelocytic leukemia cell line (HL-60) and Human lung adenocarcinoma epithelial cell line (A549) were routinely cultured in RPMI-1640 and DMEM, respectively. Medium were supplemented with 10% FBS, 2 mM L-glutamine, 100 units/ ml penicillin, and 100 μg/mL streptomycin (Invitrogen, Carlsbad, CA, USA). Adherent cells (A549) were harvested by 0.5% trypsin in Ca^2+^ and Mg^2+^-free PBS for 3 min at 37 °C. Trypsinization was stopped with the addition of fresh culture medium supplemented with 10% FBS. Cell suspensions were then centrifuged at a rotational speed of 1,000 rpm for 5 min and re-suspended at a density of 2 × 10^8^ cells/mL in fresh RPMI-1640 medium with Fluo 3-AM without FBS. After 30 min of incubation at 37 °C in a 5% CO_2_ environment, cells were centrifuged and resuspended in calcium-free Hank’s balanced salt solution (HBSS) at a density of 2 × 10^8 ^cells/mL for subsequent experiment.

### Microscopy and image analysis

Fluorescence micrographs of single cell responses were collected using a confocal laser-scanning microscope (Leica TCS SP5, Wetzlar, Germany). An argon laser (488 nm) was used to excite Fluo-3 with a 505–550 nm bandpass emission filter. Fluorescence intensities were retrieved by GenePix Pro (4.0, Axon Instruments Inc, CA, USA) and data were exported to Excel and OriginPro 8.0 for further analysis. The diameter of single cell is calculated by the measurement of the cellular area with Image Pro Plus 6.0, followed by the calculation of average diameter with πd^2^/4.

## Additional Information

**How to cite this article**: Huang, X. *et al*. Monitoring the intracellular calcium response to a dynamic hypertonic environment. *Sci. Rep.* **6**, 23591; doi: 10.1038/srep23591 (2016).
